# Supplementary material for: Hot spot prediction in protein-protein interactions by an ensemble system
Source: BMC Syst Biol. 2018 Dec 31;12(Suppl 9):132. doi: 10.1186/s12918-018-0665-8 (PMC6311905; doi:10.1186/s12918-018-0665-8)
Supplement: Supplementary file 2 — All relevant MATLAB code. A simple Matlab implement of our predictor. (ZIP 19,742 kb) [file 12918_2018_665_MOESM2_ESM.zip › Additional file 2/Read ASA/ASA Property/BID/1DDMB.txt]

 NetSurfP - Protein Surface Accessibility and Secondary Structure Predictions

  

|  |  |  |
| --- | --- | --- |
|  |  | NetSurfP - Protein Surface Accessibility and Secondary Structure Predictions  Technical University of Denmark |

  


---

```
# For publication of results, please cite:
# A generic method for assignment of reliability scores applied to solvent accessibility predictions.
# Bent Petersen, Thomas Nordahl Petersen, Pernille Andersen, Morten Nielsen and Claus Lundegaard
# BMC Structural Biology 2009, 9:51 doi:10.1186/1472-6807-9-51
#
# Column 1: Class assignment - B for buried or E for Exposed - Threshold: 25% exposure, but not based on RSA
# Column 2: Amino acid
# Column 3: Sequence name
# Column 4: Amino acid number
# Column 5: Relative Surface Accessibility - RSA
# Column 6: Absolute Surface Accessibility
# Column 7: Z-fit score for RSA prediction
# Column 8: Probability for Alpha-Helix
# Column 9: Probability for Beta-strand
# Column 10: Probability for Coil
E G  1DDMB                  1    0.847  66.683  -1.224   0.003   0.003   0.994
B F  1DDMB                  2    0.284  57.019  -2.759   0.018   0.047   0.935
E S  1DDMB                  3    0.565  66.171  -1.617   0.018   0.088   0.893
E N  1DDMB                  4    0.531  77.709  -1.437   0.018   0.088   0.893
B M  1DDMB                  5    0.223  44.582  -0.316   0.018   0.088   0.893
E S  1DDMB                  6    0.426  49.974   0.005   0.019   0.141   0.840
B F  1DDMB                  7    0.190  38.093  -0.838   0.019   0.141   0.840
E E  1DDMB                  8    0.573 100.121  -0.599   0.020   0.205   0.775
E D  1DDMB                  9    0.497  71.647  -1.538   0.019   0.141   0.840
B F  1DDMB                 10    0.181  36.347  -0.329   0.018   0.019   0.964
E P  1DDMB                 11    0.720 102.225  -1.008   0.003   0.003   0.994

Explain the output.  Go back.
```

---
